# Crystals in the community and the classroom

**DOI:** 10.1107/S1600576724000207

**Published:** 2024-02-01

**Authors:** Claire Murray, Helen E. Maynard-Casely, Ross Harrington, Stephanie McCready, Duncan J. Sneddon, Lynne Thomas, Anna J. Warren

**Affiliations:** a Diamond Light Source, Harwell Science Campus, Didcot OX11 0DE, United Kingdom; b Australian Nuclear Science and Technology Organisation, Locked Bag 2001, Kirrawee DC, Sydney, New South Wales 2234, Australia; c School of Chemistry, Newcastle University, Bedson Building, Newcastle upon Tyne NE1 7RU, United Kingdom; d Department of Chemistry, University of Bath, Claverton Down, Bath, Bath and North-East Somerset BA2 7AY, United Kingdom; Instituto Andaluz de Ciencias de la Tierra, Granada, Spain

**Keywords:** crystallography, education, outreach

## Abstract

The growing pressure on school curricula has meant crystals and the science of crystallography have been cut from or made optional for many educational programs. This omission is a serious disservice to the history and understanding of modern sciences, given that crystallography underpins many of the greatest advancements in science over the past century, is a critical component of many modern research papers and patents, and has 29 Nobel Prizes awarded in the field. This contribution describes a simple activity to target classroom and public engagement with crystallography, using marshmallows or equivalent sweets/candy to represent atoms and cocktail sticks to represent bonds, together with examples of how crystals are studied and how they are useful. Though it has a simple basis, this activity can be extended in numerous ways to reflect the aims of the demonstrator, and a few of these are described.

## Abstract 2

Most introductions to the topic of crystallography only occur during tertiary education, which presents a challenge to communicating it to wider groups. This work describes a simple activity targeted at classroom and community audiences to demonstrate crystal structure(s) and the wider impact of crystallography.

## 1. Introduction

Crystallography is just over a century old; it underpins many of the greatest discoveries of the 21st century and is directly responsible for 29 Nobel Prizes to date. It explains why water freezes, why chocolate melts on the tongue, why a drug like thalidomide can be both lifesaving and lethal, and why cement cracks in pavements; and yet it is usually omitted from secondary school curricula (Kantardjieff *et al.*, 2010 ▸) and is quickly disappearing from undergraduate courses around the world. This is a serious concern and is particularly surprising given that crystallography neatly explains or builds upon many of the mandatory elements of chemistry and physics curricula including bonding, periodicity, diffraction and waves. Additionally, crystallography deserves to be shared with the public more, given the incredible impact it has had on all corners of science and to showcase the diverse role models that the technique has fostered over its history. Indeed, crystallographers have always been pro-active in the communication of their science to many different audiences (Hodgkin, 1975 ▸; Phillips, 1979 ▸; Jenkin, 2007 ▸; Jackson, 2008 ▸).

Classroom pedagogies have long pointed towards the importance of providing opportunities for students to be directly involved in their learning experiences and to understand how knowledge (in this case about crystals) was created (Spencer, 1999 ▸). Interactive activities create a stronger personal connection to educational content, which assists in improving students’ science literacy (Heinze *et al.*, 1995 ▸). However, these activities must reflect science in a modern context, rather than as consolidated and stagnant knowledge framed as isolated facts (Stocklmayer *et al.*, 2010 ▸). For crystallography, some modern interactive practical activities based around computational modelling (Franco, 2012 ▸), construction of crystals and unit cells (Battle *et al.*, 2010 ▸; Campbell *et al.*, 2011 ▸; Collins, 2011 ▸; Casas & Estop, 2015 ▸; Ohashi, 2015 ▸; Lenzer *et al.*, 2019 ▸), crystal growing (Luft *et al.*, 2010 ▸; Rychkov *et al.*, 2014 ▸; Whelan *et al.*, 2018 ▸; Arkhipov *et al.*, 2022 ▸), and collection of diffraction data (Wilson *et al.*, 2012 ▸; Hulien *et al.*, 2015 ▸) have been reported, mainly targeting undergraduates. However, as Lenzer *et al.* report, the materials or equipment required are often complex or expensive, which prohibits their applications in high-school classrooms or in the community. Interactive crystallography education sessions for secondary students therefore heavily depend on collaboration with crystallography departments in academia and industry for access to chemicals, computers, microscopes, 3D printers and potentially even diffractometers for crystal growth, characterization and structure visualization (Grove *et al.*, 2009 ▸; Bethel & Lieberman, 2014 ▸; Gražulis *et al.*, 2015 ▸; Lumetta & Arcia, 2016 ▸).

This manuscript seeks to share a simple and widely accessible activity that communicates crystallography and can be applied to a range of audiences. The activity described builds upon this legacy of science communication to highlight the pressing need for improving crystallographic education at all levels through the use of household items that are readily affordable, familiar and easy to access. The science of crystallography is inherently three dimensional, so to convey this, we describe an activity first tested in 2012 (BCA Young Crystallographers Group, 2012 ▸). This activity has subsequently been optimized and delivered in diverse settings around the world to more than 60 000 people. It is easy to set up, can be delivered in a variety of settings and is inexpensive to deliver. We call this activity ‘The Structure of Stuff is Sweet’. Participants build their own model of a unit cell, a fundamental building block of crystallography, using familiar materials. The power of very affordable household items like sweets/candy and cocktail sticks to enable teachers and community group leaders to convey the complexity and beauty of a century-old science should not be dismissed.

This paper is organized into three subsequent sections: Section 2 where we share the basic activity, Section 3 where we describe how it can be applied in primary- and high-school classrooms, and Section 4 where we explore how it can be applied in public engagement settings. We also describe the potential hazards from the activity (necessary for risk assessments in some settings).

## 2. An accessible crystallographic activity – ‘The Structure of Stuff is Sweet’

This activity in the context of crystallography was first developed by the British Crystallographic Association’s Young Crystallographers Group, who wanted to have an interactive activity that could be used in a variety of settings. Obtaining molecular/crystal structures to demonstrate unit cells and crystal structures was expensive, so the group focused on identifying more affordable materials that could be safely used.

**Figure 1:**
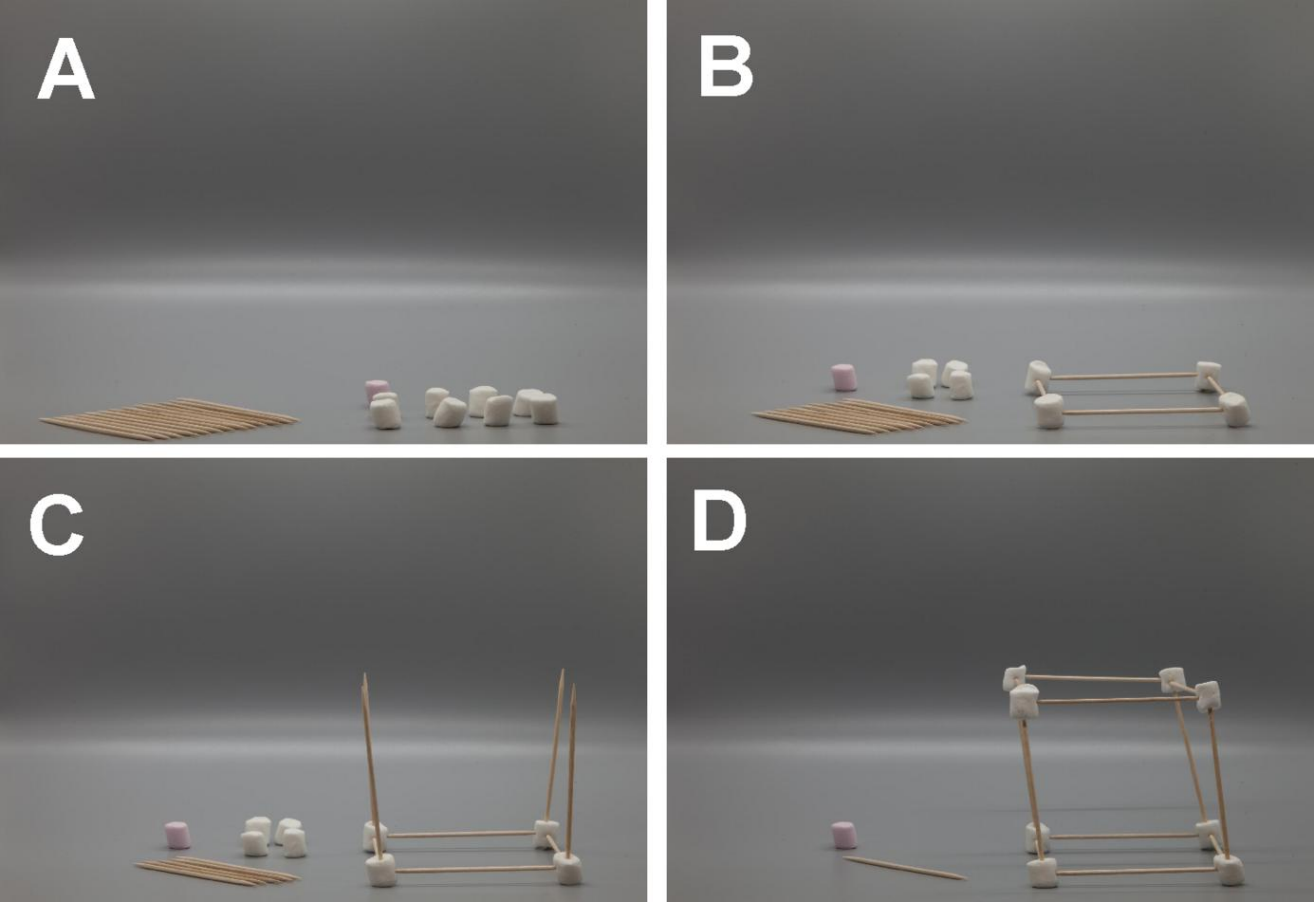
The basic equipment needed for this activity (A) with progression on how to construct a simple cubic crystal structure (B–D) – which could be a representation of the polonium crystal structure.

The basic activity requires nine sweets, 14 cocktail sticks and paper towel. By giving participants a sheet of paper towel on which to construct their unit cell, they can take their unit cell away (and eat it!) if they wish afterwards. To this end, it is also recommended to bring along a bottle of hand hygiene gel for use by participants before they take out their sweets. Fig. 1 ▸ outlines both the basic equipment needed for this activity and the construction of the crystal structure of polonium (Beamer & Maxwell, 2004 ▸).

We suggest the use of marshmallows and cocktail sticks (or truncated wooden skewers) to undertake the activity. Marshmallows and cocktail sticks/wooden skewers have long been used in various high-school practical activities, such as for the design and construction of trebuchet catapults (English *et al.*, 2013 ▸; Hudson *et al.*, 2013 ▸), highlighting brain structure and function (Foy *et al.*, 2006 ▸), building bridges (Custer *et al.*, 2016 ▸), and constructing molecules (Keller, 2011 ▸). The retention of the marshmallow’s structural integrity for enough time for it to be manipulated into position enables complex architectures such as the caesium chloride primitive cubic unit cell or the face-centred structure of sodium chloride to be constructed.

For extension to more complex structures, it is not essential to have different coloured sweets, but this does aid participants in remembering that they are dealing with different atoms. The raw materials are so inexpensive that delivering this activity is very cost effective for a small investment. Large marshmallows (∼15 cm^3^) and mini marshmallows (∼1 cm^3^) have been successfully used, so both can be deployed depending on the budget available. Gummy/gelatin sweets can be used too, although it is preferable to identify ones that have a high level of symmetry. Jelly Tots have also been successfully implemented, which give access to a wider range of ‘atomic’ colours. Vegan and halal marshmallows and sweets can be sourced from many supermarkets or online. Those undertaking this activity in Australia found the popular sweet Jubes particularly well suited to the activity. The focus in this paper is centred on sweets for two reasons: (1) this provides an overarching narrative that can run across the whole activity, which in turn supports participants to engage more easily with the content (Dahlstrom, 2014 ▸), and (2) the cost and risk assessment for these materials are relatively low. However, other materials such as beads, foam balls and marbles can be used in appropriate settings. We also provide in Table 1 ▸ some suggestions for complementary resources that (depending on the context) may be helpful.

## 3. ‘The Structure of Stuff is Sweet’ for classrooms

Crystals regularly appear in high-school practical science laboratories and within home chemistry sets, and as a result there are numerous crystal-growing competitions across the world. But it is rare that the subsequent step of explaining the context of their structure and what they consist of is undertaken. Hence, we have documented lesson plans (for high schools, supplementary file 1, and for primary schools, supplementary files 2 and 3) that describe how the basic activity of ‘The Structure of Stuff is Sweet’ can be applied in these settings. These lesson plans provide context to crystallography that can be further applied to the appropriate curricula and can be directly shared with teachers for use in their classrooms.

The learning objectives for this activity hinge around the understanding of order in crystalline materials. Accordingly, the concepts of a gas, a liquid and a solid should be introduced, especially for primary schools. For groups, this works particularly well if participants are encouraged to demonstrate what happens if they are an atom or a molecule in a confined space (*e.g.* their classroom). By decreasing the ‘atom’s’ Gibbs free energy as they firstly ‘condense’ into a liquid and then ‘solidify’ into a solid, the participants become increasingly aware of how the atoms and molecules behave in the different states of matter through their own motion. The final transition is from a solid to a crystalline solid where the group orders themselves in rows and columns facing the same direction – some guidance may be necessary from the activity leader. This creates questions about how atoms and molecules behave and why this is different for crystals compared with other solids. Returning to Gibbs free energy, it is also a convenient point to discuss how atoms will still have vibrational motion unless they are at absolute zero, and so the ordered rows and columns are incorrect unless the ‘atoms’ (participants) are in very subtle motion, *e.g.* via a constant gentle wiggle on the spot.

**Figure 2:**
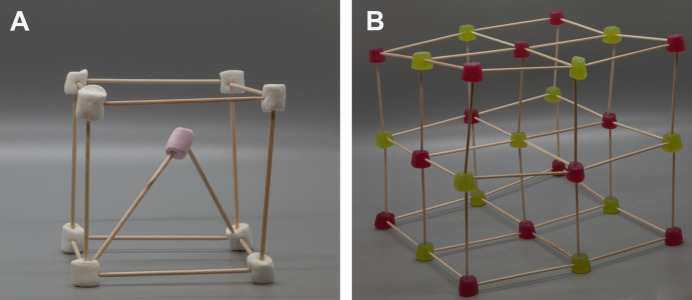
Illustration the extension of adding an additional marshmallow to the sweet crystal structure to represent a primitive cubic unit cell of a material, such as that of caesium chloride (A), and extension activity of constructing a sodium chloride structure (B).

The concepts of the components of crystallography can be demonstrated at this point by asking participants to consider what might be the smallest repeatable unit in three dimensions that could be used to represent how atoms pack in a crystal. The use of familiar stacking ‘units’ like bricks or boxes leads neatly into discussions of symmetry and some of the different crystal systems such as cubic or orthorhombic. Everyone can build a primitive cubic structure, as detailed in Fig. 1 ▸, to make polonium using 12 cocktail sticks and eight marshmallows. We noticed at this point that many participants comment on their perception of a large central void in their unit cell – this creates a clear discussion point on atomic radii, attractive and repulsive forces in molecules, and density. By using two cocktail sticks along a diagonal of the primitive cubic cell to suspend one more marshmallow, the primitive unit cell of caesium chloride can be constructed (Fig. 2 ▸). It is also possible to build the face-centred structure of sodium chloride. However, given the increased volume of materials used in this structure (14 marshmallows and 18–24 cocktail sticks), it is suggested to only get one group to make this or to construct it as a demonstration afterwards. To extend from a single unit cell to larger structures, students could construct increasingly large structures (subject to material availability) to demonstrate periodicity, or this could be collectively discussed in the classroom. An optional activity here (due to the inherent risk of using lasers in classrooms) is to bring in diffraction gratings and laser pointers to demonstrate diffraction and in turn share how we know real crystals are periodic.

For secondary education, this activity links to multiple elements in the science curricula, by providing the opportunity to explore crystals through structure and intramolecular and intermolecular bonding. It also reinforces multiple aspects of periodicity including covalent radii, ionization energies and electronegativity through the direct comparison of the CsCl and NaCl structures. This is therefore a very simple but very powerful approach to actively engaging students in their learning. In keeping with the international scope of this publication we have not specifically linked the activity to just one country’s curriculum, but instead provide an outline in supplementary file 1 describing how it could be adapted to assist in the teaching and demonstration of chemistry and other science subjects.

For primary-school educators we have provided two multi-part structured lessons in supplementary file 2 that can be comfortably delivered in two one-hour sessions. Effective delivery of these lessons does not require formal science training. A student worksheet and student guide sheets for construction of ‘crystals’ are also provided in supplementary file 3 to accompany the lessons. The science and terminology used have been simplified to suit upper primary audiences, and the conceptual learning builds appropriately to promote understanding and engagement. There is a focus on metals as they can form crystals by themselves and are familiar to primary students. Bonding is not referred to as this is overly advanced for primary learning. The hands-on activities include components of numeracy and geometry in addition to science (states of matter and materials) and allow development of critical thinking and fine motor skills.

## 4. ‘The Structure of Stuff is Sweet’ for public engagement

There are numerous settings (laboratory open days, music festivals, conferences, prior to public lectures) where the activity has been and could be used to engage small groups consisting of a range of ages and lived experiences. Assumptions of prior knowledge can be very exclusionary in a public engagement setting, but the prevalence and popularity of salt and sugar and knowledge of diamonds in daily life and culture mean that most audiences can directly relate to these crystalline materials as well as the three states of matter mentioned above. Building the activity around the idea that crystals contain a 3D ordering of atoms and molecules is therefore quite straightforward and creates a launchpad for a myriad of discussions on different types of crystals and their importance to society, as well as how we determine the structure of crystals.

**Figure 3:**
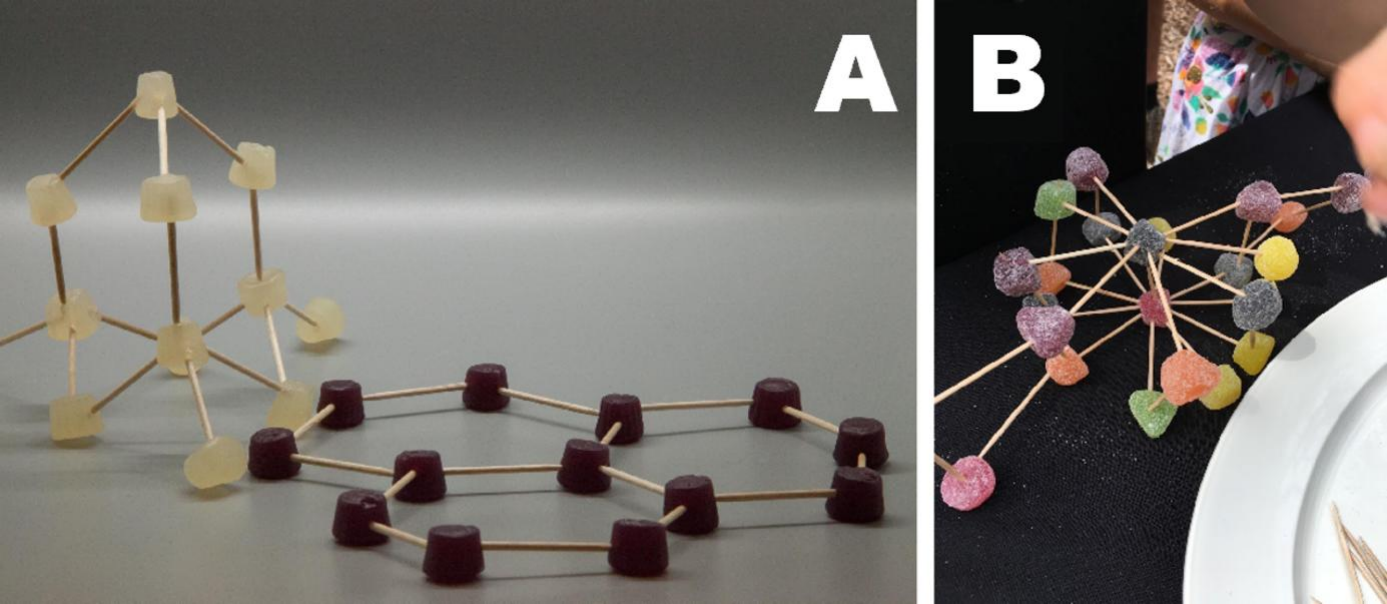
Examples of how the ‘sweet’ crystal structures can be used in a public engagement setting. In A, two representations of the allotropes of carbon have been constructed – diamond and graphite. These are both informative and tangible ways to demonstrate how the material properties of the two stem from their atomic bonding. B shows a rather inventive crystal structure put together by a young participant of the activity in Australia; this led to a discussion on the nature of bonding and stability in crystalline materials.

There are several ways to engage small groups in the activity, and here we relate a few observations/tips from conducting such sessions as part of a science festival. In such settings, it is important to realize that time to engage your audience is very limited. Preparing and rehearsing a 30 s ‘elevator pitch’ to convey why they may be interested in crystal structures, and how it is important to them, is therefore very useful. Be ready to repeat this to many groups! Ending the pitch with a question is an excellent way to intrigue the audience: ‘Do you want to make your own diamond?’, ‘Do you know that diamond and pencil lead are the same stuff?’ or ‘Do you know what is in your table salt?’ have worked well in previous events. From there, the audience can be invited to examine pre-constructed structures, either models made from sweets such as those in Fig. 3 ▸ or 3D-printed structures such as the one supplied in supplementary file 4 [alternatively researchers may wish to print their own structure; for this we refer them to Wood *et al.* (2017 ▸)]. The (now) participants can then be invited to replicate these structures and comment on the difficulty to make each one. Fig. 3 ▸ illustrates several approaches, with the potential for expansion to more complex topics such as bonding and conduction. One such example is building the graphite structure and including a representation of the delocalized electrons in the bonding that give rise to the stacking in graphite and its ability to conduct electricity. For younger audiences the freeform approach (Fig. 3 ▸B) can be very effective, and on completion can lead to excellent discussions on the nature of atoms and how complicated the symmetry of their creation is – and how it may or may not relate to real life.

The activity lends itself very well as an outreach tool for researchers in a whole range of disciplines, from those working in framework materials who want to illustrate large surface area/pores space to those in metallurgy who wish to distinguish martensite and austensite structures. Having members of the public replicate (even simply) the structures that a research group is working on can be a very powerful communication and connecting tool.

## 5. Hazards

There is potential for splinters from the cocktail sticks, which should be highlighted in any risk assessment undertaken for this work. Working with marshmallows also creates very sticky fingers, so the use of hand hygiene gel and a paper towel after the activity will aid in reducing this. Marshmallows contain gelatin, so it is advisable to contact teachers/community leaders to inform them about this in advance and to provide alternatives for those with dietary requirements or cultural restrictions from gelatin products, which may also apply to other substituted sweets.

## 6. Conclusion

To date over 60 000 people have created their own structures with this activity, and the fact that public engagement professionals are deploying it independently of crystallographers (at *e.g.* laboratory or university open days) is a clear sign of the success and the accessibility of these activities. The flexibility in the content means it is easy to modify its delivery for different audiences, and the interactive elements provide an opportunity to discuss order in crystals, which opens up further conversations around diffraction and applications of crystals in day-to-day life. This is a simple but highly effective demonstration that underpins a huge range of sciences including mineralogy, immunology, metallurgy, ferroelectrics, pharmacy and more.

## Supplementary Material

Supplementary file 1. Lesson plans incorporating the activity for high schools. DOI: 10.1107/S1600576724000207/gj5303sup1.pdf

Supplementary file 2. Lesson plan for primary schools. DOI: 10.1107/S1600576724000207/gj5303sup2.pdf

Supplementary file 3. Primary school worksheet and guides. DOI: 10.1107/S1600576724000207/gj5303sup3.pdf

3D printing file of a simple cubic structure. DOI: 10.1107/S1600576724000207/gj5303sup4.stl

## Figures and Tables

**Table 1:** Complementary crystallography resources

Resource	Citation	Resource description
CCDC outreach resources	CCDC (2023 ▸)	Activities for home learning and science festivals, including educational games
Celebrating Crystallography – An animated adventure	Royal Institution (2013 ▸)	YouTube video by the Royal Institution about crystallography
Crystallography365	Maynard-Casely & Crystallographers (2014 ▸)	Blog posts for the general public about various crystal structures written throughout the International Year of Crystallography in 2014
Diamond: The Game board game	Murray et al. (2022 ▸), Diamond Light Source (2020 ▸)	Free print-and-play board game, highlighting sciences including crystallography and scientific careers through experiments at Diamond Light Source
*From geology to biology: an interdisciplinary course in crystal growth*	Arkhipov *et al.* (2022 ▸)	Detailed list of relevant crystallography links and articles in different contexts in the supplementary information
International Year of Crystallography 2014 site	IUCr (2014 ▸)	Legacy site containing links to educational resources
RSCB-PDB educational resources	Zardecki (2008 ▸), Dutta *et al.* (2010 ▸)	Resources including activities, lessons, animations and more
*Voyage dans le cristal*	Hodeau & Guinebretiere (2015 ▸)	Travelling museum exhibition about crystallography with posters and animations
