# Supplementary material for: Crystals in the community and the classroom
Source: J Appl Crystallogr. 2024 Feb 1;57(Pt 1):181–6. doi: 10.1107/S1600576724000207 (PMC10840310; doi:10.1107/S1600576724000207)
Supplement: Supplementary file 1 [file j-57-00181-sup1.pdf]

Crystallography in the Classroom

Secondary School Students

**Learning Objective**
To develop an understanding of extended ionic and covalent structures and their resulting properties.
**Supports Prior Learning**
Difference between ionic and covalent bonding<br><br>States of Matter<br><br>VSEPR theory<br><br>Difference between a solution and a liquid
**Materials Required**
Marshmallows/sweets<br><br>Cocktail sticks<br><br>Tissues/blue roll<br><br>Hand sanitising gel<br><br>Supporting content<br><br>• Signs to aid procedure – what is a crystal, how do we study crystals, what can we learn from crystals<br>• Ball and stick diagrams of a number of structures – salt, sugar, graphite, diamond etc.<br>• Image showing difference between gas, liquid, solid and crystalline solid<br>• Diagram of unit cells of above examples<br>• Pictures of common crystals e.g. salt, sugar, diamond, crystal caves in Mexico<br>• Example image of a diffraction pattern<br>• Simple explanation of diffraction
**Suggested Supporting Content**
Diffraction grating/very fine sieve<br><br>Laser pen<br><br>3D models (e.g. Molymod) of graphite, diamond etc<br><br>Computer screen with 3D models of larger structures to rotate (3D visualisation also possible with 3D glasses)<br><br>Real samples of crystals

Steps

The aim of this activity is to explain what crystals are and how they are useful, to primary aged students and the general public. This has been done by splitting the activity into three areas:

- What is in a crystal?
- How do we study crystals?
- What can we learn from crystals?

Overall key messages

- Even though you might not have heard of it – crystals and the science of examining them are at the heart of an awful lot of important science
- The technique of crystallography was uncovered 100 years ago, and has been responsible for key scientific breakthroughs ever since
- The technique is still important today in labs and large facilities like ISIS and Diamond. We are finding ways of using crystallography to reveal the structure of more fragile and complex materials.
- Relevant in subjects from biology to space science, forensics, secrets of life!

Activity 1: What is in a crystal?

Key message for activity

- Crystals are made from repeating units of the same thing – building blocks/atoms/molecules.
- Shape of crystals depends on how the building blocks are arranged – show some different examples (salt/sugar/natural minerals).
- Different arrangements have different properties.

With this activity we are trying to explain what a crystal is, and demonstrate this by constructing crystals out of sweets and cocktail sticks.

The concept of a crystal can be introduced by referring first to solids, liquids and gases (see supporting material). A visual model could be displayed, or depending on the feasibility/number of people, the differences can be demonstrated using people moving round, each person representing an atom/molecule.

At first participants can be moving around more quickly, in random orientations, this would depict a gas. As the motion slows, participants would be able to be closer together but still moving in different directions, this would represent a liquid. Then as the motion stops, people are able to stand closer together still, but remaining in different orientations, this would depict a solid. The big difference to move from a solid to a crystalline solid/crystal is that the people (atoms/molecules) have to 'pack' themselves in ordered columns and rows, all facing the same direction. This is a crystal where the repeating unit is a person, each has exactly the same surrounding as all the others (notwithstanding size/gender/ethnicity differences).

Building on from this 2D representation of a crystal, participants can be asked to think about the smallest repeatable unit in 3D, and can begin constructing a crystal out of the sweets and cocktail

sticks. The simplest form of repeat unit is pictured in Figure 1, where all sweets at the corners are identical, this would represent a crystal of polonium. Building on to more complex 3D repeat units, two different coloured sweets can be used, this requires a significant number of sweets and cocktail sticks so may be better done as a group (Figure 2). One such crystal that exists in this was is sodium chloride, or salt. Here the chloride ions lie at each vertex of the larger cube, with sodium atoms positioned between each of them. Caesium chloride has the same make up as sodium chloride, however the caesium atom is much larger than the equivalent sodium in sodium chloride, and this causes the atoms to pack differently in the crystal. Figure 3 represents the packing in caesium chloride, here the vertex of the cube is occupied by chloride ions and the caesium resides in the centre of the cube.

In all cases these models represent a very small component of the extended structure, and their model structure is repeated almost to infinity (on the atomic scale) in 3D.

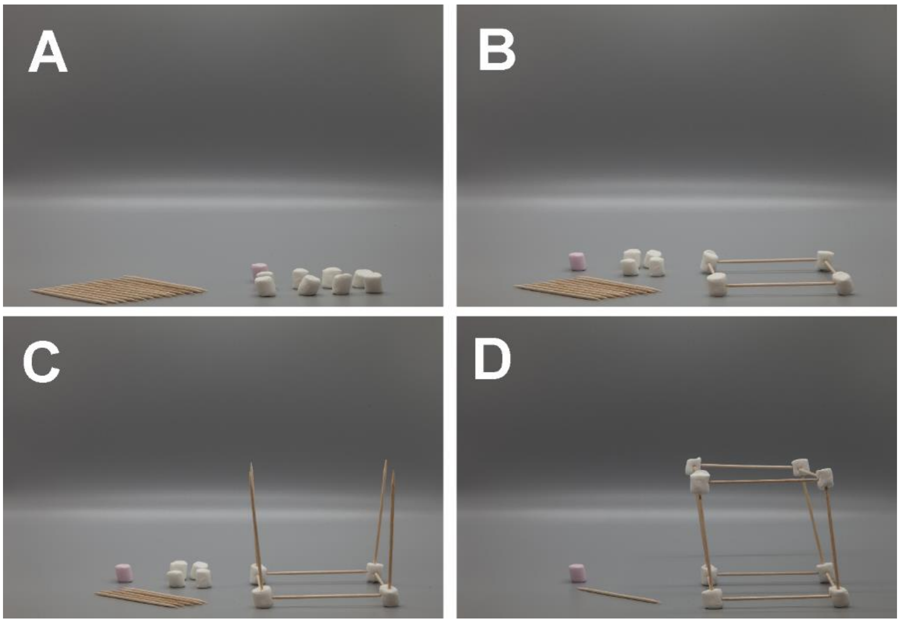

Figure 1: Smallest 3D repeating unit where all corners of the cube are the same sweet/atom/molecule. This represents a crystal of polonium.

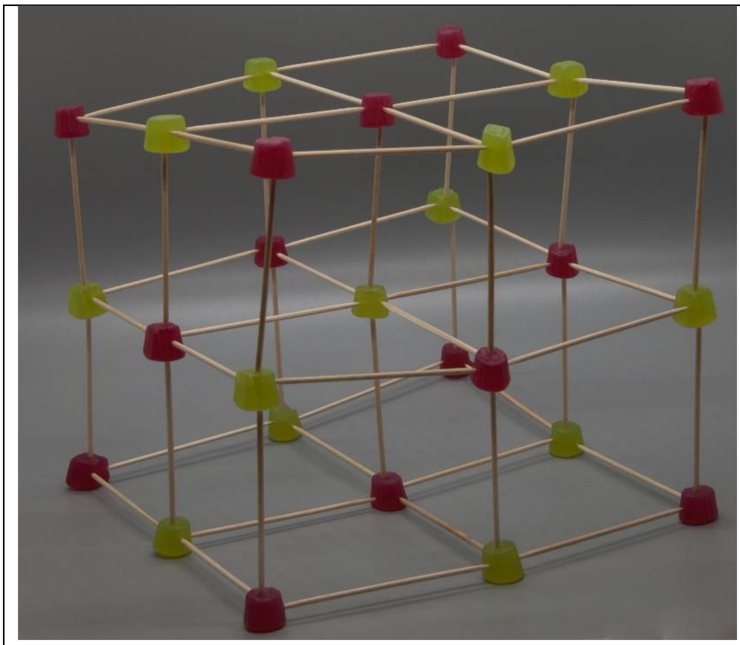

Figure 2: 3D repeating unit where two different sweets/atoms/molecules are used. This represents a unit cell of sodium chloride/salt.

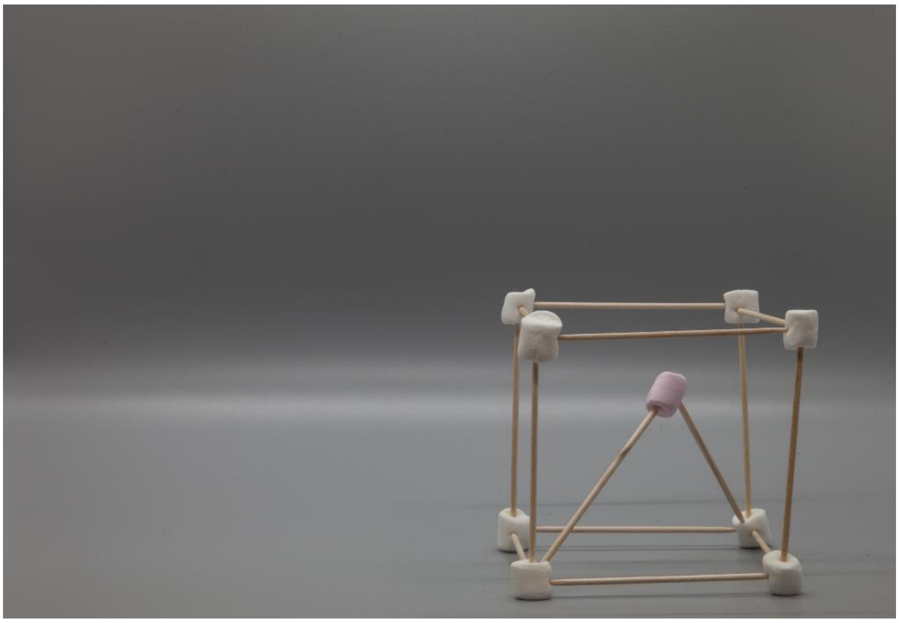

Figure 3: 3D repeating unit where two different sweets/atoms/molecules are used. This represents a unit cell of caesium chloride. Here the packing is different to Figure 2 as the caesium atom is significantly larger and resides in the centre of the cube.

Activity 2: How do we study crystals?

Key messages for activity

- Crystallographers use some incredible equipment to do their experiments.
- We look at crystals with high power X-rays, making patterns called diffraction patterns.
- These patterns tell us what the molecules/units/blocks that make up the crystal look like.

With this activity we are trying to explain the concept of diffraction and how this can be utilised to study crystals and work out the atoms/molecules that the crystals are built up from.

Diffraction is encountered in everyday life, it occurs when waves interact with some sort of matter. For example, CD or DVDs have narrow spaced lines which interact with light waves to produce the familiar rainbow pattern on the disc. Sound waves can also diffract round objects which is why you can still hear sounds round corners.

In relation to crystals, the atoms/molecules exist in very ordered rows and columns, these create planes within the crystal that waves can interact with. Due to the size of atoms, light waves cannot be used and instead X-rays have to be used as the wavelength of these is small enough to interact with the planes of the crystal. X-rays can be produced in labs, or in specially designed facilities such as Diamond Light Source, UK. Here the X-rays are fired through a crystal, the X-rays interact with the ordered structure of the crystal producing a diffraction pattern which is then recorded on a special X-ray sensitive camera.

If available, a diffraction grating/very fine sieve and laser pen can be used to demonstrate this phenomenon. The laser pen represents the X-rays and the grating the crystal. If the laser pen is shone through the grating/sieve, which has an ordered mesh of lines, a diffraction pattern is produced displaying discrete spots (Figure 4). By looking at the spots, we can work out how far apart the lines on the grating are, and also use the intensity of the spots. Crystals, which have a more complicated set of planes, produce a more complicated patter of spots, but the theory and maths are still the same. The position and the brightness of the spots can be recorded and from this we can work backwards to work out what atoms/molecules are within the crystal and how these are packed together to form the crystal.

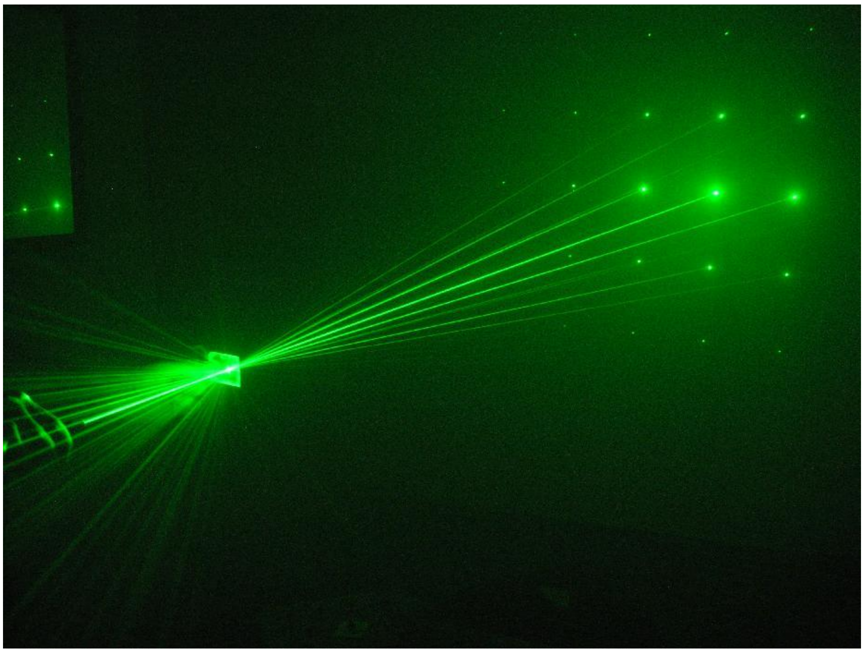

Figure 4: Image showing the effect of shining laser light through a diffraction grating. Here the light waves interact with the mesh pattern of the grating to produce diffraction spots.

Activity 3: What can we learn from crystals?

Key messages for activity

- Everything is constructed from atoms, if we can understand how the atoms/molecules are arranged it can give insight to a number of properties.
- Understanding these properties allows scientists to investigate their applications in many areas ranging from biology, chemistry, space science, materials science etc.

Carbon

Crystals of carbon exist in many different forms, the packing of these atoms in a crystal is directly related to their properties. One form of carbon is that of graphene, here each carbon atom is connected to three other carbon atoms to form flat hexagons, which build up to form hexagonal sheets. It is also possible to construct these hexagonal sheets out of sweets and cocktail sticks (Figure 5A). If a number of sheets have been made by different participants, these can be stacked on top of each other, this then creates the structure of graphite (Figure 5B), also known as pencil lead. As the sheets are stacked, but not bonded to each other, it is easy for these sheets to slide over one another. This is how a pencil works, the writing you see on the paper is these sheets being left behind on the paper from the graphite.

In comparison, diamond is another form of carbon, here each carbon atom is connected to four other carbon atoms (Figure 5C). Due to the nature of the packing of the carbon atoms it makes this form of carbon extremely strong and allows it to be use as an abrasive, for diamond-tipped drill bits and saws.This is a demonstration of how the different structures have very different properties, but without knowing the structure it’s hard to understand these properties. Having a crystal and studying the crystal with X-rays is the only way of getting the structure.

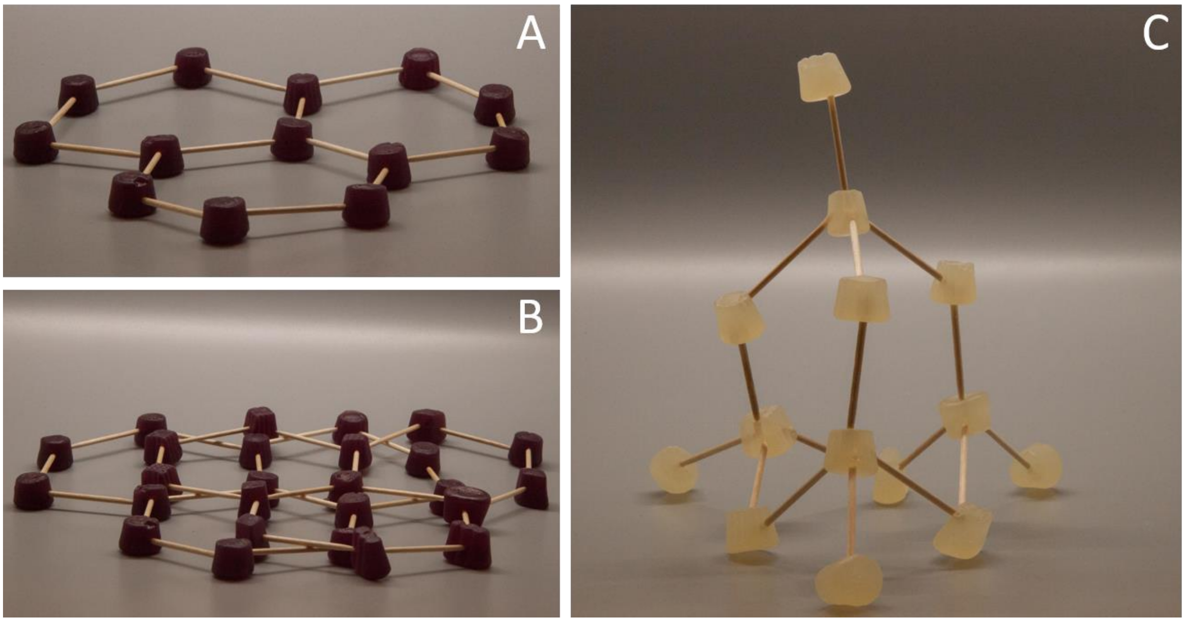

Figure 5: Representation of the ‘sweet’ crystal structures of the different forms of carbon. Panel A represents one hexagonal sheet of graphene, while panel B shows the stacking of two sheets to form graphite. Panel C demonstrates the construction of a crystal structure of diamond.

Chocolate

Chocolate is made up of tiny particles and crystals that range in diameter from 0.01 mm to 0.1 mm. These particles govern how the consumer perceives the chocolate, different arrangements of the molecules within the cocoa butter crystals govern the properties of the chocolate. The crystal structure of cocoa butter in chocolate can exist in six different forms. Form V has a superior taste and texture compared to the other forms, it snaps well and is glossy. Form VI on the other hand has a whitish coating ('chocolate bloom'), melts slowly on the tongue, and tastes coarse and sandy in the mouth. Form V can be produced by tempering the chocolate at specific temperatures; however this can degrade to form VI if left for several months at room temperature.

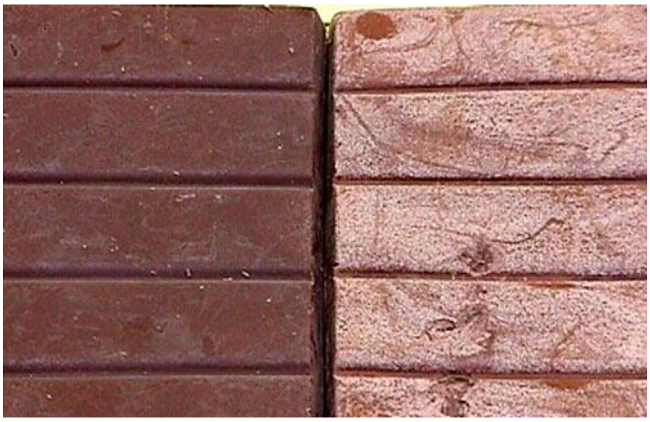

Figure 6: Form V of cocoa butter (left) and form VI (right).
https://www.zmescience.com/science/physics/chocolate-bloom-x-rays-07052015/

Paracetamol

There are two forms of paracetamol - form I is the one we take, but it is very hard and so when you try and compact it, it falls apart. Form I is the most stable and the easiest to make but because it doesn't compact, they have to add a binder to it - this is why cheap paracetamol tastes chalky (because they use chalk!) and more expensive paracetamol is smoother because they add a more sophisticated binder. There is another form of paracetamol, form II which is very hard to make, but its structure is layered so when you compact it, the layers slip relative to one another and it forms good tablets on its own. Form II is also more soluble in the blood than form I. However, form I is still used for the manufacture of tablets because form II is significantly harder to make, that there is a significant cost implication associated with it.

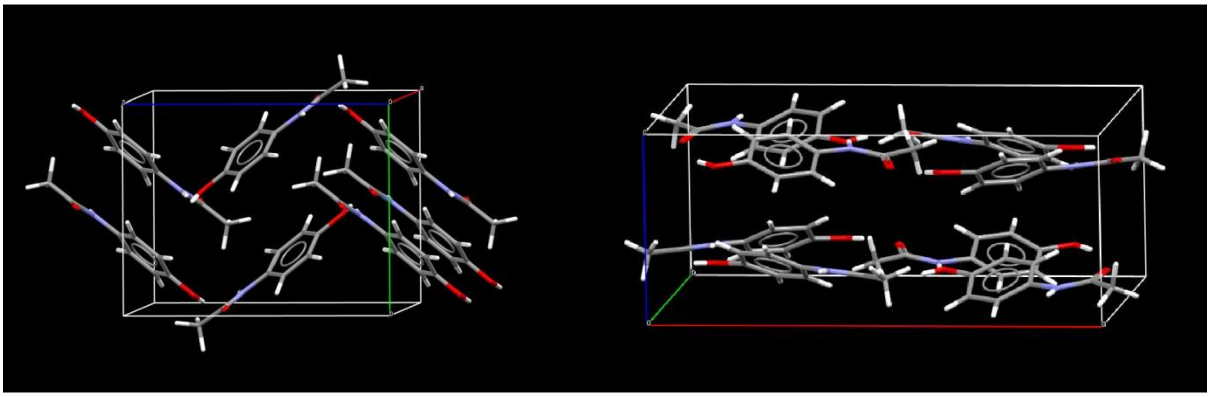

Figure 7: Two ways of packing paracetamol molecules, form I (left) and form II (right)

Thalidomide

It is possible for some molecules to be joined together in the same way, with the same number of atoms and identical connections. However, the overall 3D shape can vary, in a similar manner to a pair of hands. Your hands cannot be superimposed on each other, however if you place a mirror between your two hands, the reflection of one produces the second hand. This same phenomena can occur with a pair of molecules and is known as chirality, each molecule is known as an optical isomer.

Thalidomide was a drug used in the 1950s to treat the symptoms of morning sickness in pregnant patients. The drug worked well, however a number of the children born to mothers taking the drug were born with physical abnormalities. The location of the deformities and their severity depended on the point at which the pregnant mother started taken the medicine.

Thalidomide exists with two optical isomers (Figure 7); one treats the symptoms of morning sickness, while the other causes defects to the growing baby. The only way to truly identify the two different forms from each other is to carry out experiments to determine their crystal structures. Chemists can determine whether they have a mixture of both forms, or whether they have been able to manufacture just one of these isomers.

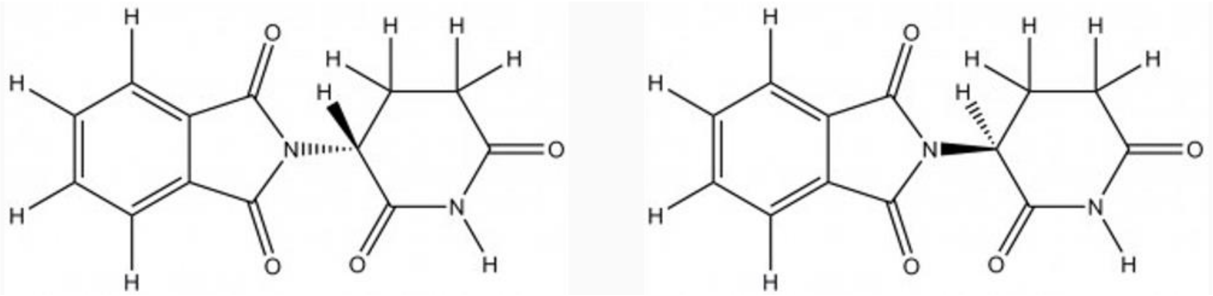

Figure 7: Optical isomers of thalidomide. The dashed and thick solid lines represent the 3D orientation of the atoms and how they differ in the two forms.

Insulin

Insulin is a small protein that our bodies produce to help us absorb glucose from the food we eat. It acts as a hormone in our bodies and if our body is unable to produce the correct amount of it, leads to diabetes. It was originally thought in the 1920's that insulin would be a rather simple molecule, perhaps similar to thalidomide, and that it would be straight-forward to first solve its crystal structure then learn how it could be synthesised to help treat those with diabetes. However, it turned out to be a large molecule (human insulin has the chemical formulae $C_{257}H_{383}N_{65}O_{77}S_6$) and the solution to its structure because one of the early grand challenges of crystallography.

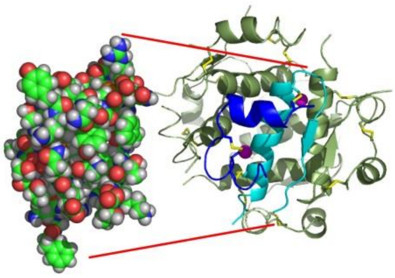

Figure 8: Illustration of the insulin monomer (left) and how it fits into the larger structure for storage

in the human body (right). By Isaac Yonemoto. - Transferred from en.wikipedia to Commons. CC BY 2.5, https://commons.wikimedia.org/w/index.php?curid=1531881

DNA

Building on the discovery of the insulin crystal structure, crystallographers became more ambitious as to the protein structures they could solve. They found new methods and strategies to solve larger and larger structures. By the 1950's one biological structure became particularly important for them to solve, that of Deoxyribonucleic acid or DNA. This is because it is this molecule that contains the genetic instructions for the development, functioning, growth and reproduction of all known organisms and many viruses. A big breakthrough in the discovery of the structure that was completed in 1953 was the diffraction pattern 'photo 51' taken by Ray Gosling and Rosalind Franklin which provided key evidence on the double helix structure.

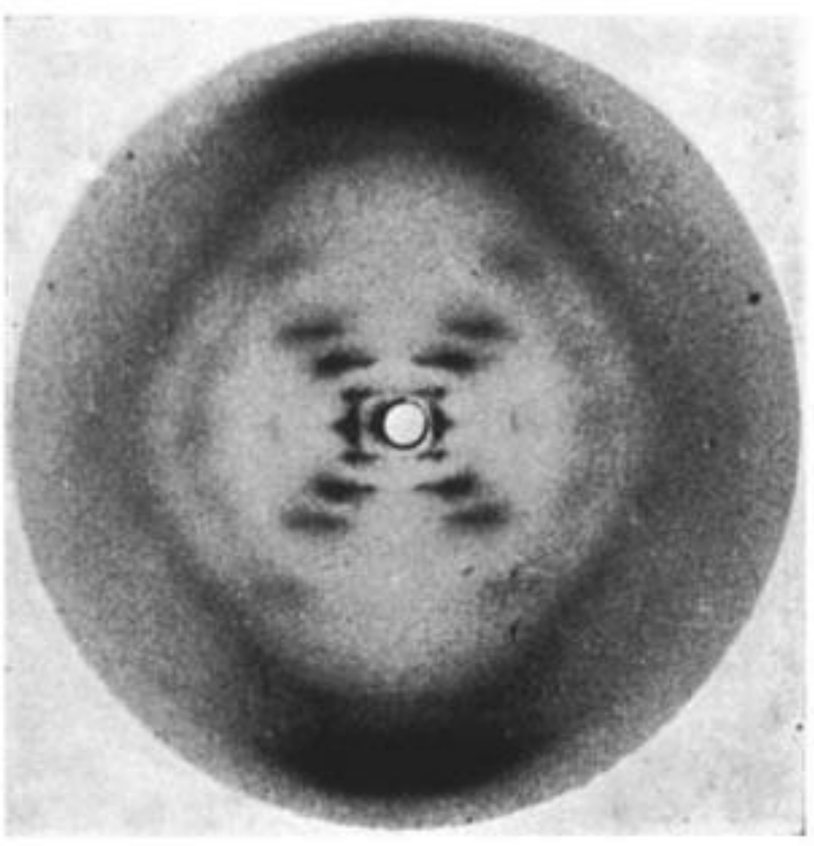

Figure 9: X-ray diffraction image of the double helix structure of the DNA molecule, taken 1952 by Raymond Gosling, commonly referred to as "Photo 51", during work by Rosalind Franklin on the structure of DNA

Teachers Notes

X-ray crystallography

X-ray crystallography is essentially a technique that relies on the same principles as using a microscope to view small objects. Here, however, we are using X-rays instead of visible light as X-rays are of a similar wavelength to the length of atomic interactions

There are two types of X-ray crystallography: single crystal and powder diffraction. Both rely on the same principles, however the amount of useful information is more limited by powder diffraction as a bulk sample is used and part of the dimension of the data are lost. This activity focuses on single crystal X-ray diffraction.

X-ray crystallography relies on the fact that X-rays interact with electrons that are concentrated around the atoms in molecules or extended structures. In order for this interaction to occur successfully, a single crystal is required. A crystal (commonly found examples are sugar and salt) is made up of a single unit (from an atom to a whole molecule) that is repeated in three dimensions to infinity (on the atomic scale), such that every 'unit' is in exactly the same local environment as every other 'unit'.

By using, the crystals act in a similar way to a diffraction grating. As the X-rays have a wave form, interactions with electron density around atoms in the crystal produce a pattern consisting of areas where the waves are in phase (leading to spots in a diffraction pattern) or out of phase (so cancelling each other and leading to blank areas). The intensity of each spot is measured by computer and collated in a file. Each data set can consist of hundreds to millions of data points, depending on the structure being investigated.

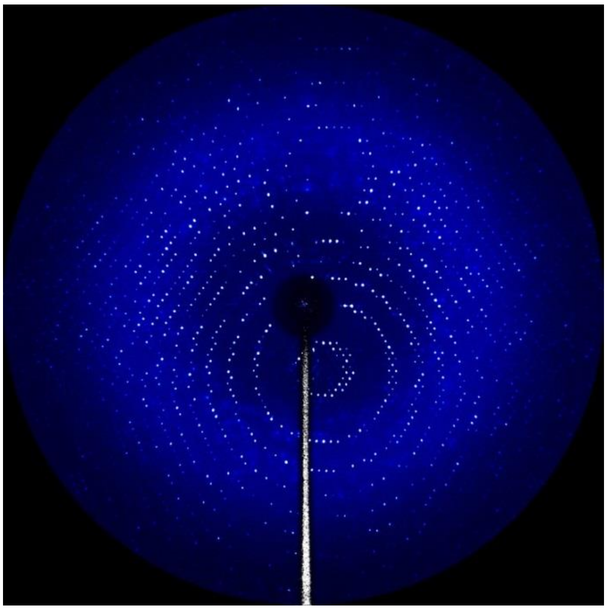

Figure 10: X-ray diffraction pattern of the enzyme glutamate. Credit: Patrick Baker. Attribution 4.0 International (CC BY 4.0)

Computing power and algorithms have advanced to such a state that where even 20 years ago collecting data and solving a structure could take 2-3 days, it is now possible in 10 minutes.

X-ray crystallography continues to be used as an analytical technique in chemical and biological research. It is also vitally important in the obtaining of pharmaceutical patents, for example in the determination of different polymorphs of pharmaceuticals; that is where molecules arrange themselves differently when forming two or more different types of crystal. Not only can his can have a significant effect on the cost of production and efficacy of pharmaceuticals, but identifying alternative polymorphs prevents competitors breaking patents on new pharmaceutical products. In addition to the importance of being able to prove the structure of any particular molecule, crystallography contributes consistently to the development of new materials for use in renewable energy, catalysis, drug delivery and CCS (carbon capture and storage).

Important contributions of crystallography:

There's a great video summary of this from the UK's Royal Institution – see it on YouTube here
https://www.youtube.com/watch?v=uqQlwYv8VQI
