# Supplementary material for: Crystals in the community and the classroom
Source: J Appl Crystallogr. 2024 Feb 1;57(Pt 1):181–6. doi: 10.1107/S1600576724000207 (PMC10840310; doi:10.1107/S1600576724000207)

Crystallography in the Classroom: Lessons for Primary Students

Lesson 1 – Introduction to crystals (55 mins)

In Lesson 1 students are introduced to crystals as being solids with an ordered arrangement of particles. They are shown diagrams, take part in a physical activity, and engage in hands-on activities that introduce two-dimensional and three-dimensional arrangements of particles. Suggested timings for each segment are provided.

1.1 Gases, liquids, solids and crystals! (15 mins)

The lesson commences with a revision of gases, liquids and solids as states of matter. The diagrams below may be used to assist explanations. It is explained that gases, liquids and solids are made up of tiny particles called atoms. We can think of atoms as having a spherical shape. They are so tiny that one million atoms can fit across the diameter of a human hair.

Gases can change shape and volume and take the shape of their containers. This is because the atoms in gases can move freely and spread out to fill the container. Liquids can change shape, but not volume, and like gases, take the shape of their containers. The atoms in liquids stay close together but can move or flow easily around one another. Solids will keep their shape when you move them or put them in a container. This is because the atoms in a solid are packed closely together so that they cannot move around. Crystals are a special kind of solid where the atoms are arranged in a regular pattern that keeps repeating itself. The external shapes of crystals are due to the regular arrangement of atoms or regular internal structure.

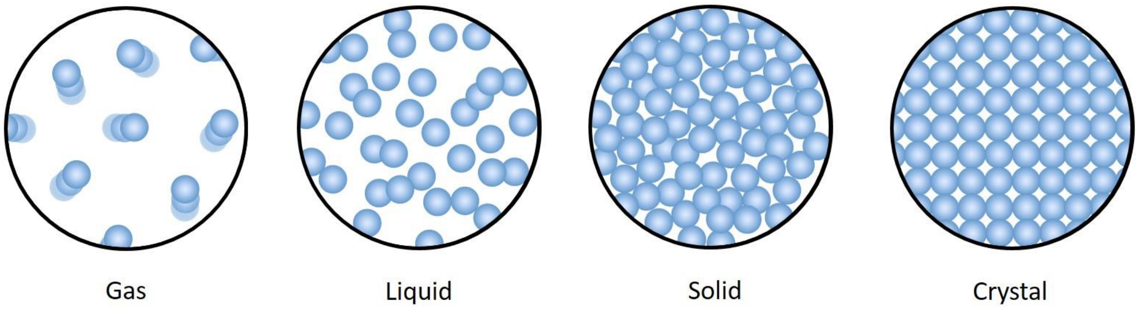

1.2. Changing state – A physical activity (15 mins)

Students pretend they are atoms in different states of matter in this activity. An open room or marked-out area in the playground is required. Pretending to be atoms in a gas, students can spread apart to fill the room and move around the room freely. When they *condense* into a liquid they must come closer together, but can continue to move around one-another. When they *solidify* into a solid, they must stand close to one another and stop moving around. It is OK if they wiggle a little on the spot, as atoms in a solid do vibrate. To represent a crystal, students must order themselves in neat rows and columns and all face the same direction.

The activity can be played as a game where the teacher calls out the state of matter and students arrange themselves quickly to represent that state. Students who make the incorrect action can be eliminated from the game.

1.3. Packing puzzle (15 mins)

Overview

A hands-on activity suitable for small groups. Students explore two different ways of arranging spheres in a single layer, then consider the three-dimensional arrangements created by adding more layers.

Resources Required

- A bag of spheres (with consistent diameter) per group. We found the following spheres (with diameters) to be suitable for this activity: polystyrene foam balls (2.5 cm), marbles (1.5 cm) or wooden beads (1.5 cm). Enough spheres are required to complete three layers as per the instructions below.
- Small square box – one per group. The box must not have rounded corners. The length should be the sum of the diameter of several spheres (at least three spheres), with additional space for half a sphere. We used boxes with an internal diameter of 8.9 cm, which was suitable for the spheres listed above.
- Student Worksheet (supplied) + lead pencil and eraser.
- Optional - Large polystyrene foam craft balls and toothpicks assembled to create three-layer models to show the class. We used 6 cm foam balls for this.

Teaching Procedure

Introduction: The activity can be introduced by asking students if they have ever helped to pack balls in a box and whether they have tried or noticed different packing arrangements. Perhaps they have observed how oranges are usually packed into a box.

Single layer: Groups are issued with an empty box and a bag of balls/ spheres and asked to arrange the spheres in a single layer (ensuring the spheres touch), following a regular pattern, and to identify two different packing methods. They draw each arrangement and determine the number of spheres that can fit in a single layer. Students then identify the sphere in the centre of the layer and count the number of other spheres it is touching. Afterwards, students are asked which arrangement fit the most spheres in a layer and why and discuss their findings. Students may be asked which arrangement they think might be favoured in crystals.

Learnings: Students identify a square packing arrangement - here the centre sphere is in contact with four other spheres. Students also identify a closely-packed arrangement in which the centre sphere is in contact with six other spheres in a hexagonal shape. In this arrangement an extra row of spheres fits in the layer. If the balls in the square packing arrangement are jiggled, they will tend to adopt a more closely-packed arrangement. Atoms in crystals prefer the second arrangement (provided they are identical and bound together by non-directional forces).

Single layer (using wooden beads) - square packing (left) versus close-packing (right):

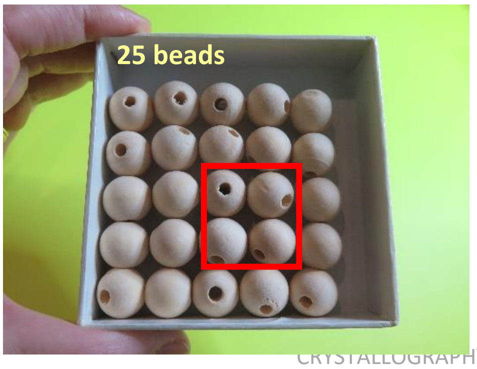

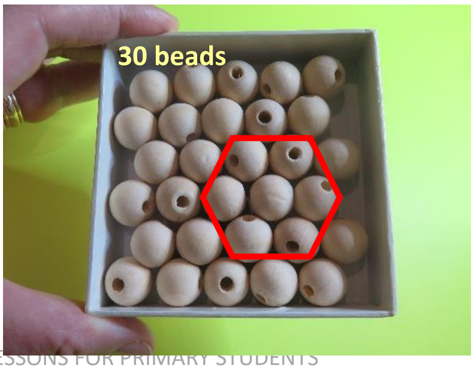

Second layer. Students gently drop one or two spheres on top of the closely-packed arrangement (to the right in photo above) and observe where they land. From this they construct a second layer.

Learnings: Students observe that the spheres in the new layer nestle in the hollows created by the first layer. After completing a second layer they may notice that half of the holes/ spaces in the first layer are now covered.

Second layer (using foam balls) – position adopted by dropped ball (left) and completed second layer (right):

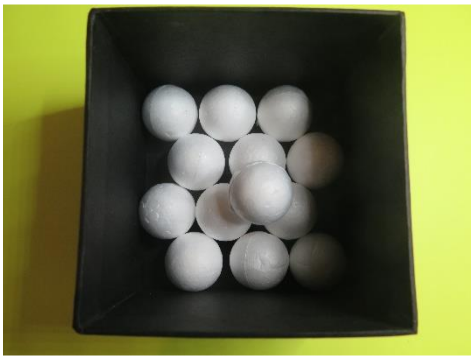

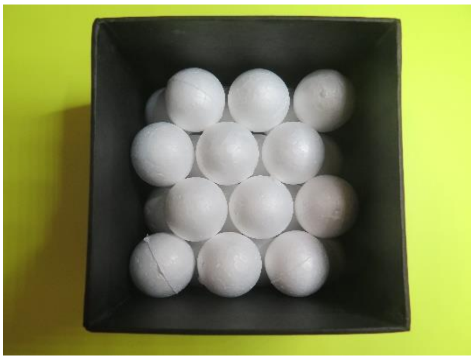

Third layer. Students are asked to create a third layer by placing spheres in the same position as those in the first layer. They may be asked to determine the smallest repeating arrangement of spheres that makes up the three layers. This is easier to determine if a transparent box is used, so that the layers can be viewed side-on (or referring to the photo below may assist).

Third layer - marbles in a transparent box:

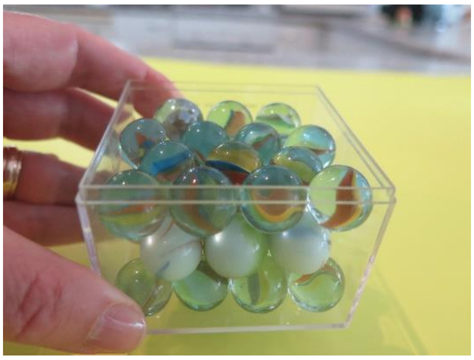

Learnings. Students learn that the repeating arrangement is two layers of hexagons (each containing seven spheres) with a triangle (three spheres) in between. This crystal structure is known as *hexagonal close-packed.*

A demonstration model for the class can be constructed by joining large (6 cm) polystyrene foam balls with toothpicks to create the three layers of this structure. This can then be compared to a model created by stacking layers of spheres in the square arrangement - a structure referred to as *simple cubic*. By comparing these two models side-on the more compact arrangement of the hexagonal structure can be readily observed by the reduction in height and reduction in internal spaces.

Third layer – demonstration models showing construction of hexagonal close-packed structure (top) and comparison of simple cubic and hexagonal close-packed structures (bottom):

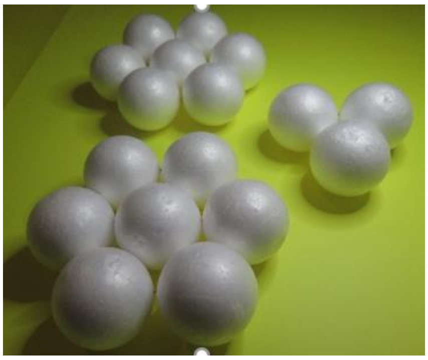

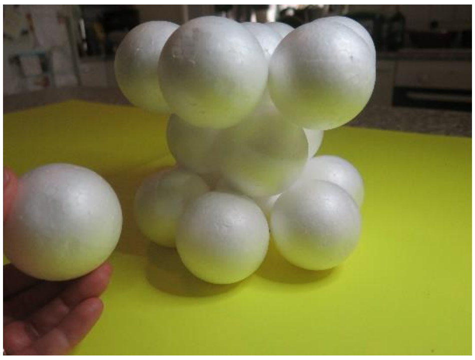

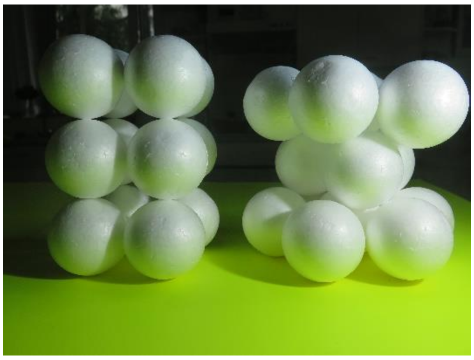

1.4. Crystals are everywhere! (10 mins)

It is explained to students that most solids are crystals and they are found in more places than we first realise. They are found in our homes as table salt, sugar and solar panels. In nature they are found at the beach and in snow as snowflakes, as well as underground as precious gemstones. Examples of solids which are not crystals are glass and plastic – here the particles are not organised in a definite pattern. Working in pairs, students are asked to research examples of crystals in nature and in their homes. They write down as many examples of crystals as they can in the space provided at the bottom of their worksheet. Their findings are discussed and simple explanations of how crystals form are provided by the teacher. Crystals can form when liquids cool, for example, when molten rock (magma) cools under the Earth's surface. Another way crystals form is when water containing dissolved salts evaporates.

Lesson 2 – Crystal structures (55 mins)

In this lesson students construct models of crystal structures with sweets and cocktail sticks and are asked to consider the stability of these structures. The lesson finishes with a short introduction to the science of crystallography and further learning can be provided with an extension activity. Suggested timings for each segment are provided.

2.1 Introduction and reflection from last lesson (10 mins)

From the previous lesson, students are asked to recount two ways of packing spheres. In the simplest method, spheres were packed in a square arrangement in each layer, so that each sphere touched four other spheres in the layer. In the second layer the spheres were placed directly on top of those in the first layer. In this arrangement, the smallest 3-dimensional repeating arrangement

was a cubic structure. (An analogy can be made to buildings and other large structures which are made up of smaller repeating structures.)

In the more closely-packed arrangement, the spheres in each layer were in a hexagonal arrangement, with each sphere touching six other spheres. The spheres in the second layer were placed on top of the holes in the first layer and the spheres in the third layer were lined up with the spheres in the first layer. In this arrangement, the smallest 3-dimensional arrangement that repeated itself had a hexagonal structure.

The atoms in crystals can follow these two arrangements, referred to as *simple cubic* and *hexagonal closed-packed* crystal structures. Crystals can be made up one type of atom or more than one type of atom. Metals are a type of crystal that we use in our everyday lives. (Students can be asked if they had thought of metals as a type of crystal in the last lesson). There are over 90 different metals and metal atoms can form crystals by themselves. Students are told that they will create their own models of metal crystals using sweets and cocktail sticks.

2.2 Simple cubic crystal structure (10 mins)

Working in pairs, students construct the simple cubic crystal structure from sweets and cocktail sweets, following the Student Guide Sheet provided. The explanation can be given that each sweet represents the centre of each atom (the *nucleus* that holds the mass of the atom, which is surrounded by space). If students finish the activity early and are waiting for other teams to finish, they can join another cube to their structure.

Resources required:

- Student Guide Sheet – Cubic Crystal Structure
- 8 mini marshmallows or gummy (jelly) sweets
- 12 cocktail sticks

Students are asked to reflect on whether there's an issue with the cubic structure. They might observe that it is a wobbly or unstable structure which easily distorts when touched. This can lead to a discussion on how diagonal cross-bracing is used in walls of buildings to add strength and help retain the square shape.

There's only one metal which has the simple cubic crystal structure, called polonium. We don't have polonium in our homes – it is rare and also very unstable.

2.3 Body-centered cubic crystal structure (5 mins)

The space inside the simple cubic structure is pointed out to students. It is explained that in crystals with a cubic structure another atom may fit in the middle of the cube, to create the *body-centred cubic or BCC* crystal structure. Working in pairs, students are asked to modify their simple cubic structure by adding another sweet following the Student Guide Sheet.

Additional resources required:

- One mini marshmallow or gummy (jelly) sweet
- Cocktail sticks – at least 2, but 4 or 8 sticks may be used.

Students are asked to consider the stability of this crystal structure and how it compares to the simple cubic structure. They should observe that this structure is less wobbly/more stable. It is explained that commonly used metals often have the BCC structure, such as iron. Iron makes up

steel, which is used in buildings and railways, and many appliances in our homes, such as, fridges, sinks, ovens, washing machines etc. Iron is a strong metal - this is due to its crystal structure! Chromium is another metal with this structure.

2.4 Hexagonal close-packed crystal structure (20 mins)

Students are told that they will include three layers in this structure: two hexagonal shapes with a triangular shape in between. Working in pairs, students construct the *hexagonal close-packed or HCP* structure following the Student Guide Sheet.

Resources required:

- Student Guide Sheet – Hexagonal Close-Packed Crystal Structure
- 17 mini marshmallows or gummy (jelly) sweets
- 27 cocktail sticks for construction of the three layers
- 6 short skewers (12 cm long) to join hexagons (6 cocktail sticks may be used if skewers are not available – this produces a shorter structure)
- Additional 6 cocktail sticks to join centre triangle from top and bottom (when using skewers). If cocktail sticks are used to join the hexagons, the centre triangle can only be joined from the bottom of the structure (requiring 3 additional cocktail sticks).

The explanation is provided that HCP is a common crystal structure for metals, as atoms of the same type like to pack close together. An example of a metal with this structure is titanium. It is a lightweight and strong metal that is used in aircraft and implants for our bodies! Titanium is often referred to as the wonder metal of the 21st century.

A discussion may be included on how hexagon shapes are often found in nature (beehives, turtle shells, fly eyes, bubbles joining together) as they can pack closely and are also strong shapes.

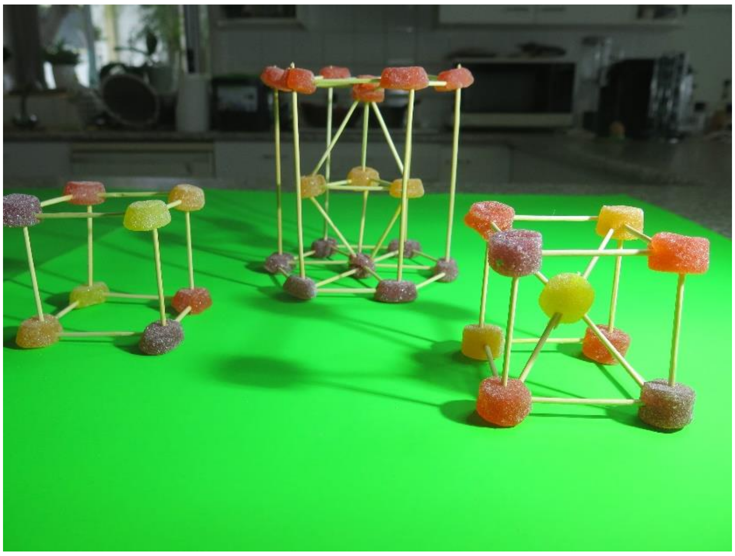

Crystal Structures (L to R): Simple Cubic, HCP, BCC

2.5 How scientists research crystal structures (10 mins)

Students may wonder how scientists are able to work out the structures of crystals. (Even the most powerful light-focussing microscopes can't see single atoms!). This can lead to an introduction to the important science of *x-ray crystallography*. A simplified introduction is provided below.

Scientists called *crystallographers* can work out the positions of atoms in a crystal using X-rays. When X-rays are fired at a crystal they will scatter to produce a pattern which is detected as a two-dimensional image with a special X-ray sensitive camera. As we know, crystals have a regular internal structure. The X-ray pattern created will depend on the arrangement of the different layers or planes of atoms in the crystal, and each crystal structure therefore gives a different X-ray pattern. Scientists can apply mathematics and geometry to convert the two-dimensional X-ray pattern to a three-dimensional model of the crystal showing the locations of the atoms.

Crystallography leads to a better understanding of many areas of science that affect our lives including the study of metals in engineering structures, proteins in our bodies, starches in our foods, medicines that make us well, plus many more important applications.

Optional activity. Working in pairs, students shine a torch on one of their crystal structures (created above) to produce a shadow on a large piece of paper placed underneath the structure. They then trace the shadow with a pencil or marker pen. Sunlight can also be used to create the shadow. The shadow drawings are collected by the teacher, shuffled and handed out. Students try to identify which crystal structure matches the shadow drawing they have been given.

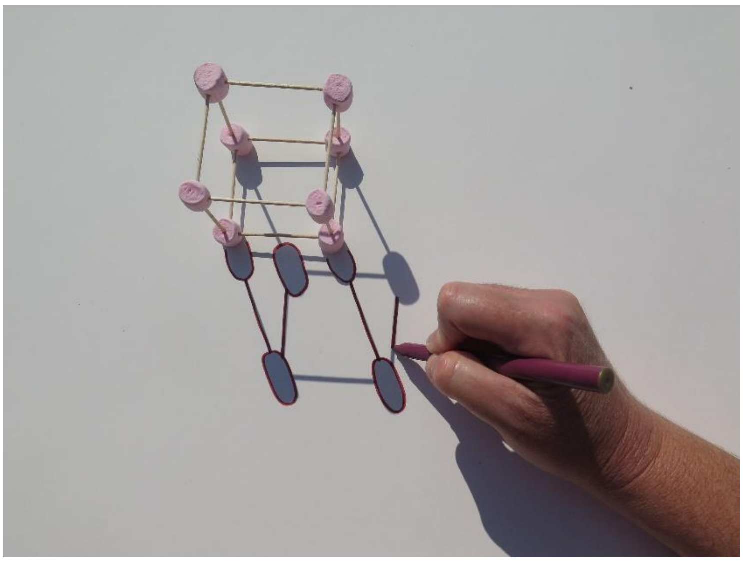

2.6 Extension activity – investigating other crystal structures

Students can research other crystal shapes and choose one to study further, for example, trigonal, monoclinic, orthorhombic, tetragonal or triclinic structures. They can describe the shape and find an example of a crystal with that structure. They can draw or make a model of the crystal. They can list the atoms it contains and any interesting information about their chosen crystal.

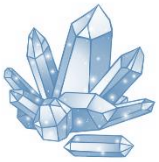

Supplement: Supplementary file 2 [file j-57-00181-sup2.pdf]
