# Supplementary material for: Crystals in the community and the classroom
Source: J Appl Crystallogr. 2024 Feb 1;57(Pt 1):181–6. doi: 10.1107/S1600576724000207 (PMC10840310; doi:10.1107/S1600576724000207)

Student Worksheet – Packing Puzzle

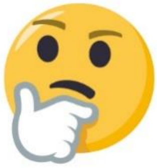

Explore two different ways of arranging spheres in a single layer inside a box.

1. Using a pencil, draw the different arrangements (as viewed from the top):

2. Count the number of spheres in each box and write these numbers in the spaces below.

Total number of spheres =______ Total number of spheres = ______

3. Determine how many spheres are in contact with a sphere in the centre of the arrangement and fill in these numbers below.

No. of spheres touching centre sphere = ______ No. of spheres touching centre sphere = ______

4. For each arrangement determine the simplest shape which repeats and draw a line around this shape.

5. List examples of crystals in the space below.

Examples of crystals I can think of:

____

____

____

____

Student Guide Sheet – Cubic Crystal Structures

Simple Cubic Crystal Structure

Materials needed:

- 8 mini marshmallows or gummy (jelly) sweets
- 12 cocktail sticks

Procedure:

1. Construct a square with 4 sweets and 4 cocktail sticks
2. Repeat, to give two squares
3. Join squares, one on top of the other, using the remaining 4 cocktail sticks.

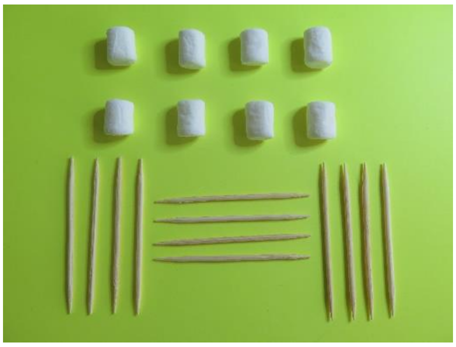

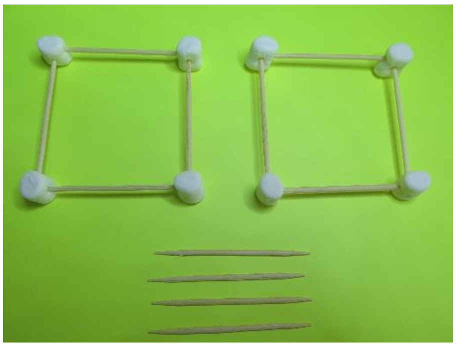

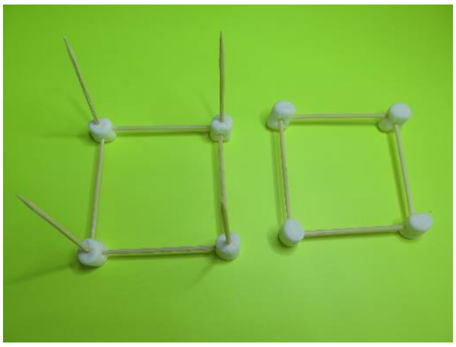

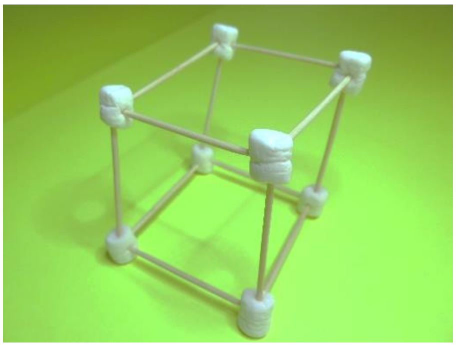

Body-Centered Cubic Crystal Structure

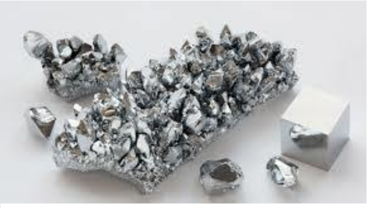

Chromium crystals: Wikipedia

Additional materials needed:

- 1 mini marshmallow or gummy (jelly) sweet
- 4 cocktail sticks

Procedure: Attach a sweet to the centre of the simple cubic structure using 4 cocktail sticks.

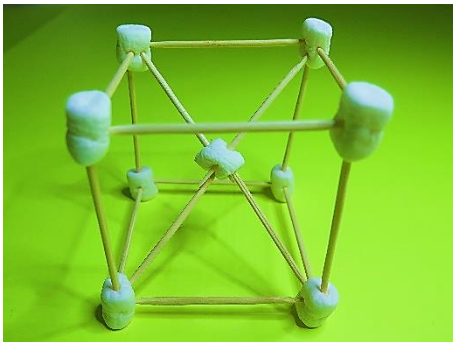

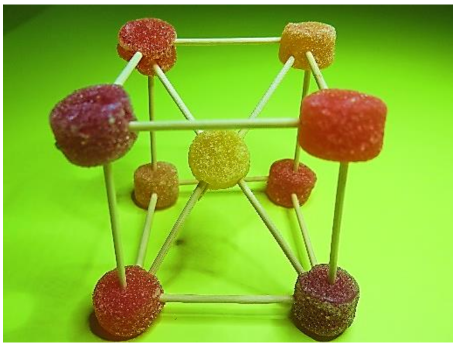

Student Guide Sheet – Hexagonal Close-Packed Crystal Structure

Materials needed:

- 17 mini marshmallows or gummy (jelly) sweets
- 27 cocktail sticks to create 2 hexagons and 1 triangle
- 6 short skewers (12 cm) or cocktail sticks to join hexagons
- 3-6 cocktail sticks to join middle triangular layer

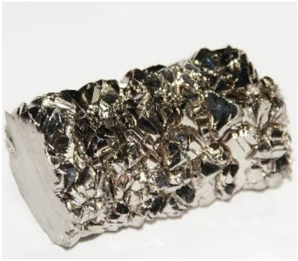

Titanium crystals: Wikimedia Commons

Procedure:

1. Construct a hexagon with 7 sweets and 12 cocktail sticks
2. Repeat, to give two hexagons
3. Construct a triangle with 3 sweets and 3 cocktail sticks
4. Join hexagons, one on top of the other, using 6 skewers or cocktail sticks
5. Insert triangle inside the hexagonal prism
6. Join triangle in centre of hexagonal prism using 3 or 6 cocktail sticks

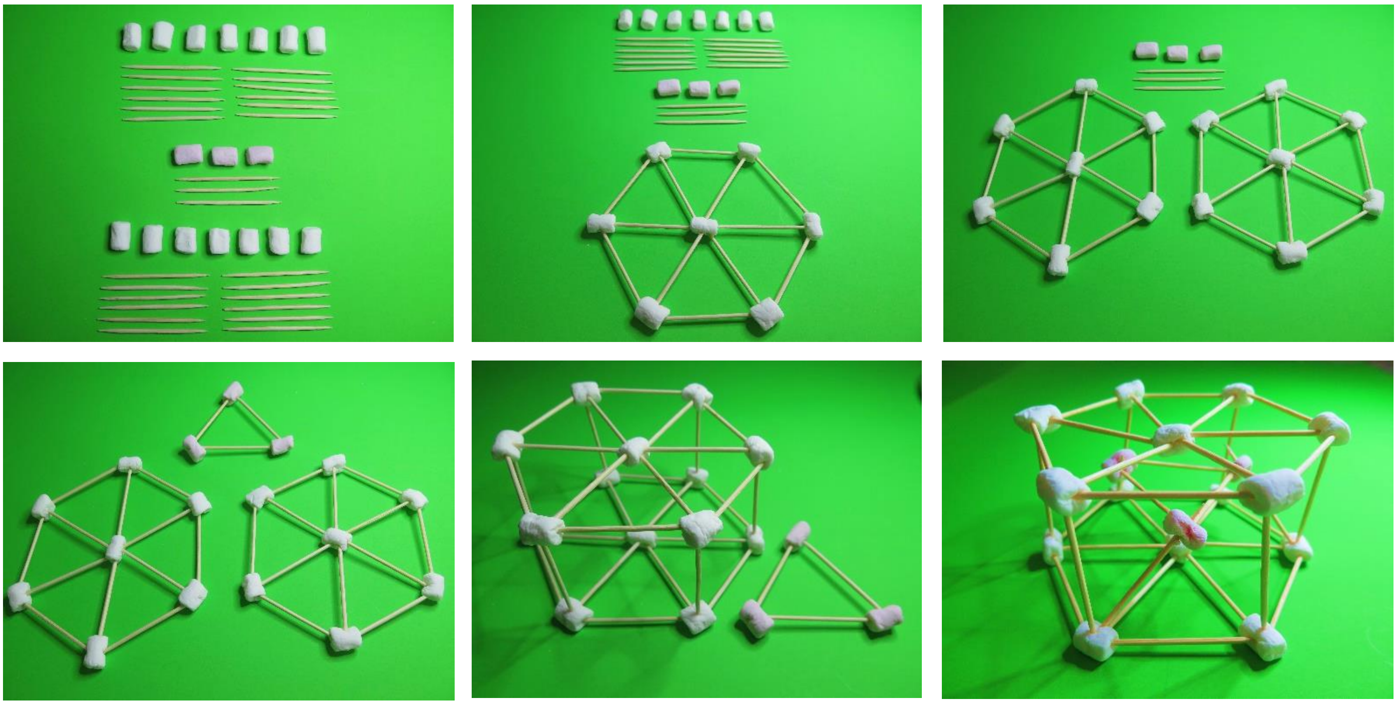

Hexagons joined with cocktail sticks (left and centre) and skewers (right):

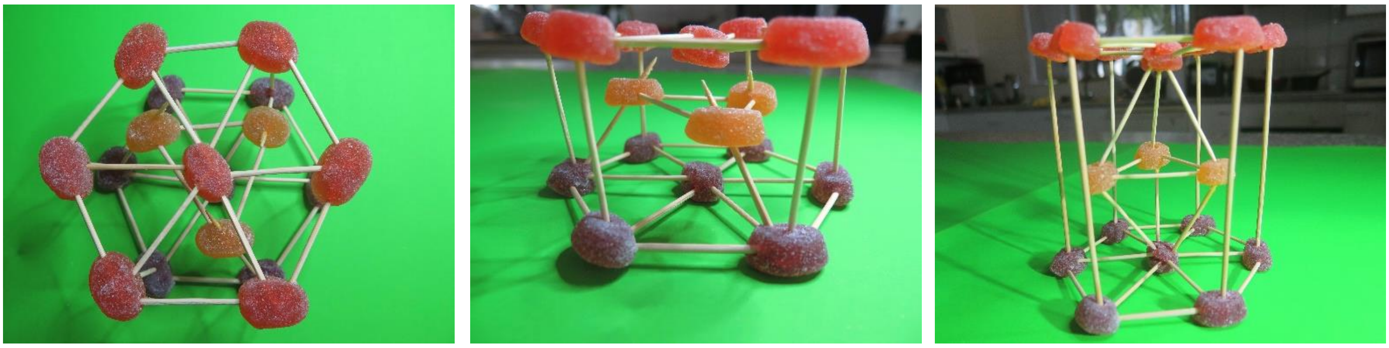

Supplement: Supplementary file 3 [file j-57-00181-sup3.pdf]
